# Attitudes towards Bachelor-level education amongst Bavarian midwives: Results of a descriptive cross-sectional survey in Bavaria, Germany

**DOI:** 10.18332/ejm/100558

**Published:** 2018-12-22

**Authors:** Bernd Reuschenbach, Andrea Komlew

**Affiliations:** 1 Catholic University of Applied Sciences, München, Germany

**Keywords:** Germany, midwifery training, university qualification, academic midwife, Bavaria

## Abstract

**INTRODUCTION:**

In Germany, the traditional vocational midwifery training lasts three years. This training is marked as Qualification Level 4 of the European Qualification Framework (EQF). There are very few midwives with a Bachelor degree, and an even smaller number of universities that offer a qualification program at EQF Level 6 (Bachelor). The aim of this paper is to analyze the attitude and interests of traditionally educated German midwives in undertaking a university degree.

**METHODS:**

Midwives were surveyed within the framework of a descriptive cross-sectional study. The survey has 13 items and was available as a web-based survey between 1 November 2016 and 31 January 2017. Alternatively, the midwives were able to send a hardcopy version of the questionnaire back by mail, anonymously. Data was obtained from 534 midwives. The quantitative data were analyzed using SPSS (Version 23). The qualitative data were structurally analyzed using a qualitative content analysis.

**RESULTS:**

It was found that 57.7% of the surveyed midwives are interested in completing a university degree, and 40.1% could imagine themselves pursuing a degree. The respondents were overwhelmingly positive in their opinion of university qualifications and qualification upgrades. Nevertheless, the open-question responses indicated that there are strong concerns surrounding the level of proficiency and experience-based knowledge taught for a university degree, which are essential elements in the traditional training system.

**CONCLUSIONS:**

Although midwives understand the importance of a Bachelor degree, they need to be informed about the expertise and practical skills taught in the degree curriculum.

## INTRODUCTION

The demand for higher-education level midwives appears to be picking up momentum worldwide^1^, with a variety of education programs and standards. In Germany, the majority of midwives are being trained in specialized vocational training colleges to an EQF Level 4 standard^2,3^. This qualification level is low compared to the neighbouring German-speaking countries, Austria and Switzerland, which have established an EQF Level 6 standard training level. Germany is the only country in the European Union which offers non-university courses^4^.

There are 58 midwifery training schools in Germany. Each school is affiliated with an obstetric hospital and is led by a medical director as well as an instructor of midwifery practice (midwifery educator).

The standard course for a German midwife trainee lasts three years and consists of 1600 hours of theoretical training and 3000 hours of practical training. Currently, there are around 21000 midwives in Germany, with 800 midwives completing the training annually^5^. Both the theoretical and practical content are provided through the midwifery training school and are regulated by the German training and examination regulations for Midwives (‘Ausbildungs- und Prüfungsverordung für Hebammen, 1987’) and the German Midwifery Law (‘Hebammengesetz, 1985’). The practical training involves attendance in the delivery suite, postnatal ward, neonatal intensive care unit, in the operating theatre, and surgical and medical wards. The students receive remuneration for their training^6,7^.

**Figure 1:**
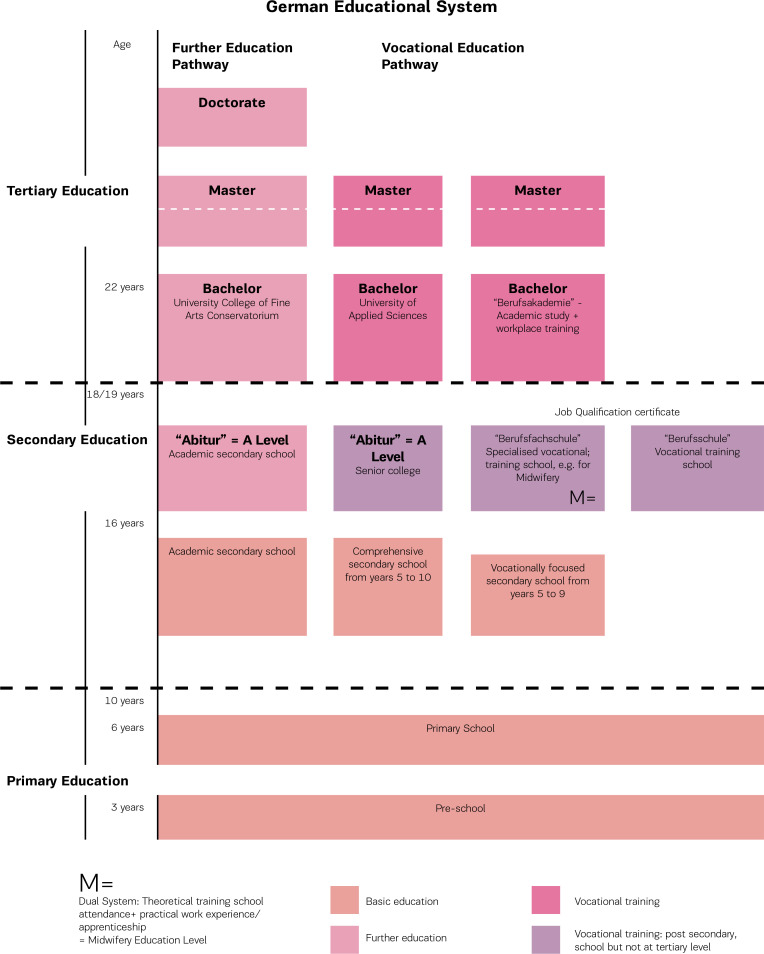
German Educational System (translated and modified from http://mavoieproeurope.onisep.fr/de/files/2012/11/allemagne_de.jpg)

Only applicants with an ‘A-Level’ course completion (which consists of 8 or 9 years of secondary school) are accepted into the training program. Midwifery training in Germany belongs to a traditional, German-evolved dualprofessional training system^6^. The training schemes within this system are already embedded in the secondary education level (EQF Level 4) and not at university level. The institutional separation of vocational and university training has become entrenched over the last 200 years and has complicated the transition between the non-academic professional training system and academic training in the university system (Figure 1). Although Germany is well know for its practice-orientated professional training system, the health science professions are disadvantaged with regard to having a ‘privileged profession’ in the EU^8^. Hence the reason why midwifery training in Germany is facing a restructuring.

The German Midwifery Association has promoted midwifery university programs for many years. This promotion is a result of the changing requirements for which traditional training is having difficulties fulfilling^9^.

One major reason why the qualification levels are changing is the increasing and changing demands of women and newborns. There has been a global shift to a ‘holistic’ approach that incorporates the supportive and preventive needs required in addition to the typical maternal and newborn care^10^. Thus, the task spectrum required of midwives has become more complex, and the pattern of professional practice has changed. Midwives are now expected to guide women throughout the whole reproductive phase. They are required to support preventative health care during pregnancy, birth, the postnatal period and the child’s first year of life^11^. Despite the changing demands in the modern-day required support, it was found in a study across 56 German midwifery training schools that there are structural training deficiencies in the program^3^. It was found that the curriculum, development and quality of training had been neglected over a long period. The traditional training, which is regulated by a law passed in 1987, only prepares participants for hospital-based care.

Consequently, certain aspects such as early intervention, care in the domestic environment, education and counselling have been scarcely acknowledged or taught^11^. The German Association of Midwives has promoted, for decades, restructuring the training, and advocated that the training standards be elevated to EQF Level 6. In so doing, the German Association of Midwives follows the recommendations of the European Association of Midwives that also sets the minimum standards of midwifery education and practice within the EU.

As a result, the training would meet international standards and encompass all the changing requirements in the professional area^12^. The first Bachelor courses in Germany started in 2008. The EU Directive 2013/55/EU passed on 17 January 2014 (Directive 2013/55/EU of the European Parliament and Council, 2013), was a large step towards initiating the qualification upgrade process.

The Directive, effective in 2020, requires all midwives to finish twelve years of education (e.g. Gymnasium in Germany) before they are allowed to begin their training or degree. Subsequently, Germany established the first university programs for midwives in the country, adapting the model provision from the professional legislation of 2009 (law for the adoption of a model provision in the professional legislation for midwives, speech pathologists, physiotherapists and occupational therapists, 2009).

Currently, there are thirteen Bachelor degrees and one Masters degree offered for midwives in Germany^13^. The university programs have varying structures. In one program, the university is completely responsible for the theoretical training, in another there are cross-over models in which both the university, as well as the ‘Berufsakademie’ (vocational college of advanced education), are responsible for the training. Additionally, there are also university programs available for midwives who have already received the vocational qualification and wish to obtain the university degree (a qualification upgrade program). The duration of the university program varies between two years, for postqualification programs, and up to four years, if the university is responsible for the whole qualification.

At present, the proportion of midwife university graduates to the number of midwife training school graduates is still small.

In Germany, most of the university programs that exist for midwives are situated in the northern states, and they are programs of universities of applied sciences. In Bavaria (a southern state), for example, there are no specific midwifery university programs, but it has always been possible for midwives (with 12-year education) to study pedagogics or management.

Bavaria is the second largest German state in population, with about 12.8 million residents, and is the largest state regarding land area^14^. There are currently around 3000 midwives, as well as eight midwifery training schools in Bavaria. The local midwives’ association in Bavaria has been promoting, for many years, the need to upgrade qualification levels to university degrees. Despite their efforts, there has been no success to date^15^.

The attitude and position of the German Association of Midwives with regards to the demand for a university degree for midwives have been made clear. Nonetheless, the views of the midwives themselves have, until now, been left unpublished. The information resulting from this study could be relevant for the implementation of new course programs and methods to increase the acceptance of higher qualifications in the practice field.

This study aimed to analyze the attitude and interest of midwives in Bavaria towards university qualifications.

The research questions posed were: ‘How do midwives in Bavaria value a midwifery university qualification? Could the midwives envision themselves studying such a course?’ and ‘Which focus areas do midwives deem as especially important in such a degree?’.

## METHODS

The research questions were posed using a descriptive cross-sectional survey. We aimed to reach every midwife in Bavaria. The methods and results are described with respect to the Checklist for Reporting Results of Internet E-Surveys (CHERRIES) and the STROBE-Guidelines ‘Strengthening the Report of Observational Studies in Epidemiology’^16,17^.

The questionnaire was distributed by the Bavarian Midwives’ State Association, in November 2016, to all of their members. According to their facts, 86% of midwives in Bavaria (e.g. 2850) are members of this association.

In an accompanying letter, the participants were informed that they could fill out the attached questionnaire or complete it online. The letter also informed the participants about the legal and ethical aspects of the study; it included a description of the study objectives, the length of the survey (13 items), the average time required to fill out the survey (2–3 minutes), and the process for opting out of portions of the questions.

### Questionnaire

The topics in the questionnaire were developed collaboratively with midwives, midwifery research professors, colleagues from institutional review boards and experts from other universities. The questions were tested, analyzed and then adapted via multiple pre-tests. The questionnaire was reduced from 22 original items to 13 items during the pre-test phase. Questions concerning the preferred study topics were excluded in the end, as a means to improve the response rate.

Furthermore, the wording of the different study structures was adjusted to improve comprehension for people without any university experience. The user-friendliness and technical functionality of the electronic questionnaire were also tested before the questionnaire was released. There was no randomization of items or adaptive questioning, meaning all participants received the same version. The thirteen surveyed items were distributed over seven webpages. Respondents were able to review and change their answers using a ‘Back’ button. The E-Survey was available online between 1 November 2016 and 31 January 2017. Alternatively, the midwives were able to send the hardcopy version back through the mail, anonymously.

The questionnaire (both the online and hardcopy version) consisted of closed questions with a three-level answer scale and a semi-open question format. At the end of the questionnaire, the respondents had the opportunity to express their own opinion regarding the issue of ‘qualification upgrade’. The open questions at the end of the survey were:

1. Do you find it meaningful for midwives to complete a university qualification?
2. Can you imagine beginning a midwifery university qualification yourself?
3. Which focus areas are particularly important to you in a university qualification?
4. Is there anything else which you would like to share with us regarding the issue of midwifery university qualification and about this questionnaire?’

The quantitative data were analyzed via descriptive statistics using SPSS (Version 23). The qualitative data were analysed following the procedure recommended by Mayring^18^. The answers received by the respondents were inductively aggregated to core concepts. An iterative procedure was used to finalize the main and underlying concepts, and new sentences were used to confirm the preliminary concept.

Supplementary sociodemographic data were also requested, such as: age, work experience, level of school education completed, and current professional duties.

### Ethical aspects

Participants were assured in the questionnaires (both written and E-Survey), that all information and records would be kept confidential, that participation was voluntary, and that the collected data were not be used for any purpose other than for this study. There was no hidden IPaddress check and no other means to retrospectively trace the identity of the participants. A non-personalized link to the online survey was enclosed in each letter. The survey was open and was not password-protected. There was no obligation to answer all the questions. The online survey was located at www.soscisurvey.de, which is a non-profit German company which services E-Surveys. The website provider is well-known in Germany, with high-level data protection standards (i.e. SSL encryption and a secure server environment), including no IP-Recording, no IPChecking, no cookies or any other log file analyses, as a means to provide maximum user privacy.

## RESULTS

After three months of data collection, the E-survey received 842 visitors, and 396 completed surveys. In all, 138 hardcopy versions of the questionnaire were returned through the mail, and 544 questionnaires were completed. Ten questionnaires were omitted due to a lack of responses or unrealistic statements. Therefore, the total number of analyzable datasets (N=534) corresponds to a participation rate of about 18.7% of the midwives who were sent a letter. There was no significant difference between the online and paper-back versions concerning the sample or the results of the questionnaire, so the data from both questionnaire versions were merged. Participants who took the E-survey needed on average 165 seconds (SD=96) to finish the questionnaire. The completeness rate (percentage of items answered) was 89% (SD=4.6).

### Sociodemographic data

The sociodemographic data indicated that the mean age of the participants was 40.1 years (SD=10.5) and the average job experience was 18.8 years (SD=10.3) (Table 1).

**Table 1:** Sociodemographic parameters of the sample

	*%*	*N*
**Age (years)**		
18-28	16.3	87
29–38	28.1	150
39–48	28.7	153
49–58	23.2	124
59–68	2.6	14

**Total**		528

Range		18 – 68
Mean		40.1
SD		10.5
**Job experience (years)**		
1–5	9.7	52
6–10	14.0	75
11–15	14.4	77
16–20	17.0	91
21–25	12.7	68
26–30	12.2	65
31–35	7.5	40
36–40	3.7	20
41–45	1.5	8

**Total**		496

Range		1–45
Mean		18.8
SD		10.3

Out of the 544 submitted questionnaires, 512 were completed by midwives and 20 by midwifery students (Table 2). We found no substantial or statistical differences between midwives and midwifery students. Therefore, we aggregated the data of both groups. Most of the midwives were found to have an ‘A level’ certification, meaning a secondary school certification after 12 years of schooling (Table 2).

**Table 2:** Qualification and occupation (N = 496)

	*%*	*N*
**Formal education qualification**		
‘A Level’ secondary school certification (post 12 years schooling)	46.8	250
Advanced technical college entrance qualification	15.2	81
General certificate of secondary education (post 10 years of schooling)	24.5	131
Certificate of secondary education (post 9 years schooling)	0.9	5
University degree	9.9	53
Other	1.3	7
**Occupation**		
Independent midwives (without obstetrics)	41.9	224
Employed plus freelance work	34.3	183
Employed	12.2	65
Students	3.7	20
Midwifery educator	3.4	18
Maternity leave	7.5	40
Unemployed	0.7	4

### Quantitative results

After the data were analyzed, it was shown that 57.6% (n=309) of the respondents found it meaningful for midwives to aspire to a university qualification (Table 3), and 25.2% (n=135) were undecided. Similarly, it was found that 42.0% (n=216) of the participants could imagine undertaking a midwifery university qualification themselves and 17.1% (n=88) were undecided.

**Table 3:** Results concerning the motivation to study and attitudes to upgrade the qualification

	*Agree*		*Neither agree nor diagree*		*Disagree*			
	*%*	*N*	*%*	*N*	*%*	*N*
**Motivation**								
In my opinion, it is meaningful to aspire to a university qualification	57.6	309	25.2	135	17.2	92		
I can imagine undertaking a midwifery university qualification, e.g. to study after the vocational training.	42.0	216	17.1	88	40.9	210		
**Would you be willing to reduce your working hours for a university degree if you were able to study right after the vocational training?**								
I am willing to stop working completely during the university qualification	4.1	18						
I could imagine reducing my working hours by 75% during the university qualification	16.0	70						
I could imagine reducing my working hours by 50 % during the university qualification	52.2	288						
I could imagine reducing my working hours by 25% during the university qualification	15.1	66						
I am not willing to reduce my working hours during the university qualification	12.6	55						
	***Very important***		***Important***		***Somewhat Unimportant***		***unimportant***	
	***%***	***N***	***%***	***N***	***%***	***N***	***%***	***N***
How important is it for you to work and study at the same time?	56.6	246	35.2	153	6.9	30	1.4	6

Furthermore, the majority of the respondents (52.2%, n=228) could imagine reducing their own working hours by 50% in order to achieve a university degree, 4.1% (n=18) were prepared to give up their work completely to do the qualification and 12.6% (n=55) could not imagine reducing their workload at all. Subsequently, the majority of midwives (56.6%, n=246) rated the ability to continue to work whilst undertaking a degree as extremely important, and slightly more than a third (35.2%, n=153) rated it as important.

The midwives were also asked to indicate which focus areas they would prefer most during their university qualifications. Table 4 shows six different program choices at the thirteen existing German universities offering midwifery degree programs. The respondents were asked to indicate which course content they found to be the most important. The majority of the midwives and midwifery students preferred the thematic areas: ‘Professional practice in midwifery tasks/practice’ (72.3%, n=386) and ‘Academic work’ (56.4%, n=301). Just under half of the midwives requested the course ‘Business financial fundamentals’ as part of the qualification (47.6%, n=254). ‘Pedagogical content’ was important for 39% (n=208), followed by ‘Ethical aspects’ for 31.8% (n=170). Nearly a quarter of the midwives (23.2%, n=124) were interested in the topic ‘Early intervention’ as a means to increase their familiarity with the practice area of community midwifery. In total, 44 people (7%) made further responses. Some of the midwives indicated their preference for the physiology of pregnancy, birth and the postpartum period with a salutogenic approach. Others wanted psychological content in the university qualification, especially about breastfeeding, bonding and the fundamentals of psychologically appropriate communication in dealing with women and their families.

**Table 4:** Preferred focus for the university qualification

*What are focus areas important to you in a university qualification?*	*N*	*%*
Professional practice in midwifery tasks/practice	386	72
Academic work/midwifery research	301	56
Business financial fundamentals/management	254	48
Pedagogical contents	208	39
Ethical aspects	170	32
Early intervention/community midwifery	124	23
Other	41	7

### Qualitative results

At the end of the questionnaire, the midwives and midwifery students were able to write statements regarding their opinion of midwifery university qualifications. In total, 135 respondents took advantage of this opportunity. The information provided revealed a glimpse into the opinions of Bavarian midwives towards the qualification upgrade process. Two core concepts from the feedback provided by the midwives were revealed using the content analyses procedure^17^:

1. Benefits for the profession
2. Threatening the practical competencies

1. Benefits for the profession

There were 42 people who made positive statements to the core concept above, e.g.

‘This is an excellent thing, in my opinion, which is connected with the public ‘appreciation’ of our profession.’ (Case number 265).

Many women stated that the new qualification would improve the attractiveness of midwifery:

‘I believe that a midwifery university qualification is overwhelmingly meaningful! For one thing, to mould the profession more attractively and, for another thing, to collaborate with the doctors equitably in the everyday setting. The profile of our job should be re-defined or more exactly defined; the differentiation in the care of pregnant women as a fundamental focus theme in midwives’ hands, supporting physiological births and comprehensive psychosocial care in the post-natal period should be emphasized in the university program.’ (Case number 537).

2. Threatening the practical competencies

Despite the positive feedback received regarding the concept of ‘benefits’, there was also much feedback concerning the lack of connection with practice. Several participants felt that the traditional care of pregnant women was threatened if a university qualification replaced the vocational training course.

‘I find a midwifery university qualification for our professional standing superfluous. I see myself as being in a profession which is pre-occupied overwhelmingly with the practical ‘care’ in a clear area. Ultimately, I don’t need an academic background. I need, of course, a lot of knowledge and experience in assisting birth, caregiver’s understanding and a motivated application of my knowledge… . Sometimes I have the feeling, that through the discussion over the necessity of a midwifery university qualification, we lose sight of the actual ‘goal’ (the woman during the pregnancy, during the birth and afterwards… . What do I actually want to achieve with a university qualification?’ (Case number 665).

Other midwives stated that it isn’t possible to develop expertise in midwifery through purely theoretical lessons. They believe it is more useful to gather expertise in practice fields and that women should have a chance to achieve this practical expertise without a university degree.

‘I find it absolutely not good, that in the future there will only be midwives with an A-level certificate or with a university program. What about all the practice, the handholding, the personal relationship with the women?? How can that be learnt when one sits in the University? I don’t have an A-level and I am happy that I have managed to complete my training without a university qualification. I have my absolute dream job, and I am overjoyed every day to be able to practice this profession. Others who have not had the chance to become midwives and didn’t have an A-level certificate – this chance will be absolutely destroyed! And I find that simply sad! A midwife who has been to university and had an A-level certificate is in no way a better midwife!’ (Case number 623).

In 29 comments, cautionary references were made in which the subjects proclaimed that the professional uniqueness of midwifery should not be threatened by a university qualification. Midwifery practice was described in the comments as a ‘craft’ that is promoted fundamentally through good quality and practical training.

‘I also think, that the overhaul of the training of midwives in Bavaria is a great necessity, especially regarding the theoretical part in order to adapt to the European standards and to improve quality. The establishment of a university program indeed satisfies this aspect. (…) Nonetheless, I have the worry that the practical aspect will lose its prominence in training, as this is certainly the more important…’ (Case number 887).

‘(…) I believe that the midwifery profession is principally a CRAFT! First one must understand his craft, and I find that, to date, this aspect falls far too short at the universities. Theoretical knowledge is indeed available, but the practice simply turns out differently. In that respect, we midwife fresh out of the midwifery training school were already prepared for this. I cannot imagine, how a university qualification can pass on the practical fundamentals?’ (Case number 338).

## DISCUSSION

According to the actual development, the German Ministry of Health aims to establish university education for all midwives. Nonetheless, the views of the midwives themselves have, until now, been left unpublished.

It was found in this study that the majority of Bavarian midwives and midwifery students support midwifery university qualification. Almost half the respondents were prepared to undertake a university degree. Midwives and midwifery students indicated their preferences for profession-specific content as a part of the university qualification. The retention of the physiological aspects and the attention to the original midwifery duties is revered. Midwifery is interpreted as an experience-based ‘Art’ and ‘Craft’ that develops through practical experiences. This aspect of the profession should be kept and highlighted in the university qualification.

Worldwide there is growing evidence that supports the necessity of Midwifery in healthcare, and the positive effect it has on women and newborns^1,10^. Nonetheless, there has been a ‘system-levels shift’ from maternal and newborn care to skilled care for all, including preventive and supportive care^10^. This shift demands new skills and higher levels of qualification, in particular for midwives.

Compared to other European countries, midwifery training in Germany is a unique program. The traditional course method is to take part in a vocational training school program, which corresponds to an EQF Level 4 certification. Nevertheless, there have been increasing developments towards a transition to university training. The midwives’ associations throughout Germany are promoting this transition phase, but are running into concerned perspectives of the midwifery training schools and the traditionally trained midwives. The future role of the midwifery training schools with respect to the university programs remain unclear and the question as to whether or not there will be a separation between the areas of practice for midwives with EQF Level 4 and midwives with EQF Level 6 remains unanswered. The insecurities with regard to the qualification upgrade were also clear in the open answers from the questionnaires corresponding to this paper. The dual system in Germany, which places a large focus on practical training, has led to a high evaluation of competency and practical performance in previous years. Evaluations in other countries showed that such vocational training with an intensive mentoring program is relevant to learning practical competencies^19^. Insufficient practical experience during a university program would be disadvantageous in developing a professional identity^20^. Hence the reason why the dual system has been complimented worldwide for its practical orientation. Nevertheless, midwifery training through a vocational school results in lower-than-wished qualification levels, which are inappropriate for current requirements. The majority of the midwives only know this system and find it hard to understand the added value of a university qualification. As a result, there are very few midwives with Level 6 qualifications, and therefore also fewer role models in the facilities and in the development of innovative care models. Additionally, midwives feel that their ‘craft’ is being threatened and are unable to judge the advantages of a university qualification over the current vocational training for the women in their care and themselves.

All of these aspects are to be evaluated until 2020 in the model university program currently being implemented. The merits of academically qualified midwives compared to EQF Level 4 qualifications were insufficiently researched in the past because the difference does not exist in other European countries. In all other European countries, a Bachelor degree is already the standard course.

In comparison, in the area of nursing there are many studies that support the argument for a university degree. Goode et al.^21^ state, for example, that nurses with academic qualifications are more skilled in critical thinking, and exhibit a higher level of professionalism and leadership qualities. In addition, the psychosocial competence and communication ability of Bachelor degree qualified staff was assessed to be higher. Midwives require all these skills as well, as a result of the changing requirements and their autonomous status. Reflective practice and critical thinking are competencies that are strongly promoted throughout the academic training^22^.

In the nursing field, there are a lot of studies that demonstrate positive outcomes for patients under the care of nurses. The studies show that the higher the education of the nurses, the safer the patient^23^. There is a lack of such studies in the midwifery field, as most countries have only one qualification level so that there is no possibility to compare the effects of different education levels of midwives.

The ever-changing needs and complex management of women and newborns demand the development of highlevel education for midwives, hence why many countries are paving new paths in education and developing competencybased Bachelor Midwifery programmes^24,25^. Furthermore, with the growing number of university programs, midwifery research will expand. Despite that midwifery research is done by midwives in Germany since 1980, until now most research is done by obstetricians with their favoured pathological and somatic orientation. The new system-shift in the midwifery field demands a special midwifery research approach^26^. The upgrade of education and more specialized programs for midwives will foster professional identities and the development of the profession in Germany.

### Limitations

The survey, of course, cannot reach every Bavarian midwife. The low response return of 18.7%, indicates that the selective effect may have occurred in both directions. It could be that critics or supporters felt especially addressed by the survey. Furthermore, the online survey tended to appeal to younger generations, amongst whom it has already been confirmed that they have a greater affinity for university training, thus potentially over-valuing the actual appraisal. Nevertheless, despite receiving a remarkably fewer number of hard-copy questionnaires, the responses were similar to the responses in the online survey.

Moreover, there was no access protection to the online survey. Thus, it cannot be ruled out that the same midwife participated in the survey more than once. This potential bias is an inherent consequence of the methodology. We decided not to use a personalized link to the online survey to ensure high participation rates and high standards of anonymity.

## CONCLUSION

Despite the small sample, the survey reflected the opinion spectrum of the Bavarian midwives and midwifery students. The survey is important for helping to determine, on the one hand, the number of university places in the future and, on the other hand, the best possible concepts (regarding content and structure) for the restructuring. Conclusively, there were words of warning in the free text at the end of the questionnaire from those that feel the origins of the midwifery profession to be in danger. The professional association still has a lot of work to do in convincing and informing all stakeholders to participate in the qualification upgrade process. Nevertheless, despite the critical tendencies of a few, the majority of midwives and midwifery students are ready for an elevation of the training level to the tertiary sector. The course for university training in Germany is set.
